# Working Memory Training and Cortical Arousal in Healthy Older Adults: A Resting-State EEG Pilot Study

**DOI:** 10.3389/fnagi.2021.718965

**Published:** 2021-10-21

**Authors:** Chiara Spironelli, Erika Borella

**Affiliations:** ^1^Department of General Psychology, University of Padova, Padova, Italy; ^2^Padova Neuroscience Center, University of Padova, Padova, Italy

**Keywords:** working memory, older adults, resting state, EEG, cognitive resources, transfer effects

## Abstract

The current pilot study aimed to test the gains of working memory (WM) training, both at the short- and long-term, at a behavioral level, and by examining the electrophysiological changes induced by training in resting-state EEG activity among older adults. The study group included 24 older adults (from 64 to 75 years old) who were randomly assigned to a training group (TG) or an active control group (ACG) in a double-blind, repeated-measures experimental design in which open eyes, resting-state EEG recording, followed by a WM task, i.e., the Categorization Working Memory Span (CWMS) task, were collected before and after training, as well as at a 6-month follow-up session. At the behavioral level, medium to large Cohen's *d* effect sizes was found for the TG in immediate and long-term gains in the WM criterion task, as compared with small gains for the ACG. Regarding intrusion errors committed in the CWMS, an index of inhibitory control representing a transfer effect, results showed that medium to large effect sizes for immediate and long-term gains emerged for the TG, as compared to small effect sizes for the ACG. Spontaneous high-beta/alpha ratio analyses in four regions of interest (ROIs) revealed no pre-training group differences. Significantly greater TG anterior rates, particularly in the left ROI, were found after training, with frontal oscillatory responses being correlated with better post-training CWMS performance in only the TG. The follow-up analysis showed similar results, with greater anterior left high-beta/alpha rates among TG participants. Follow-up frontal high-beta/alpha rates in the right ROI were correlated with lower CWMS follow-up intrusion errors in only the TG. The present findings are further evidence of the efficacy of WM training in enhancing the cognitive functioning of older adults and their frontal oscillatory activity. Overall, these results suggested that WM training also can be a promising approach toward fostering the so-called functional cortical plasticity in aging.

## Introduction

Working memory is the ability to retain and simultaneously manipulate information for use in complex cognitive tasks (Miyake and Shah, 1999), which is considered one of the core basic mechanisms of cognition (e.g., Gamboz et al., 2009). Not only it is involved in various skills, including everyday life functioning (Borella et al., 2017a), but it is also among the factors accounting for age-related differences across the life span. Indeed, working memory (WM) has a clear and linear change. i.e., decline, with aging (e.g., Park et al., 1996, 2002; Borella et al., 2008), which is accompanied by anatomical and neuromodulatory changes, as well as alterations in functional brain activity patterns in older adults (i.e., Reuter-Lorenz, 2000; Raz, 2005; Reuter-Lorenz and Sylvester, 2005). These findings have encouraged a growing interest in developing WM training procedures to slow down or attenuate age changes in the WM performance of older adults. In particular, WM training is aimed not only at improving information-processing systems of individuals but also at inducing changes in how individuals process information, through more flexible use of their own resources (e.g., Borella et al., 2019b; Carbone et al., 2019). Therefore, not only is WM training is theoretically expected to provide benefits in the trained WM tasks (so-called specific training gains) but also to give improvements in such a core cognitive mechanism would also produce general effects in untrained cognitive abilities related to it (so-called transfer effects) (e.g., Borella et al., 2017b). According to recent reviews and meta-analyses (i.e., Karbach and Verhaeghen, 2014; Teixeira-Santos et al., 2019; Hou et al., 2020), WM training for healthy older adults has been shown to provide large (Karbach and Verhaeghen, 2014; Teixeira-Santos et al., 2019) and endurable (Hou et al., 2020) training gains in tasks similar to those trained. However, less consistent conclusions have been reported regarding the generalizability of WM training benefits: improvements to untrained tasks, i.e., transfer effects, usually are weaker than training gains are (Karbach and Verhaeghen, 2014), with mixed and less endurable effects (Teixeira-Santos et al., 2019; Hou et al., 2020), although some exceptions have been found (e.g., see Borella et al., 2017b, 2019a).

Among the different training procedures adopted with older adults, to our knowledge, the WM training program proposed in the study by Borella et al. (2010) is the only one showing promising results. Their training produced short- and long-term specific transfer benefits (see Borella et al., 2017b), even extending to tasks related to everyday life (Carretti et al., 2013; Cantarella et al., 2017; Borella et al., 2019a). Furthermore, it is one of the few procedures that other laboratories have adopted (Brum et al., 2020) and whose results have been replicated. The benefits of this WM training approach are considered to be due to: (i) the training timing (every 2 days), which provides sufficient time to consolidate the skills the participants acquired (see Borella et al., 2010); (ii) the procedure used, which is adaptive, meaning that participants are trained at a level of difficulty coming close to the limits of their own capacity; (iii) the tasks, which are always novel and challenging, thus engaging different cognitive processes and sustaining participants' interest and motivation (Borella et al., 2010, 2017b).

Despite the interest in WM training, little is known about how these WM cognitive interventions affect the structure and functioning of the older adult brain (see Nguyen et al., 2019, for a review). This is quite surprising given the well-documented association between the neurobiological and functional brain changes occurring with increasing age, particularly within the prefrontal cortex, and performance on cognitive tasks involving WM or generally the executive functions. Compared with other neuroimaging approaches, electroencephalography (EEG) is particularly suited for studying maturational brain changes because it is a non-invasive technique that can be used to directly measure the cortical functioning of a human brain (e.g., Anokhin et al., 1996; Rossini et al., 2007). EEG also provides reliable measures that are important both for assessing neural correlates of healthy aging and detecting functional neural changes from healthy to pathological aging, both at rest and when executing tasks (see Rossini et al., 2007, for a review). Changes in the frequency of resting-state brain oscillation, such as a decrease in posterior alpha power, have been associated, for instance, with altered cerebral blood flow and cognitive functioning in older adults with dementia, as compared to those with healthy aging (Rossini et al., 2007). Furthermore, both cognitive and/or physical training significantly affect neural oscillations (e.g., Styliadis et al., 2015; Klados et al., 2016; Reis et al., 2016), as well as event-related potentials (ERPs) (e.g., Spironelli et al., 2013; Zendel et al., 2016) in older adults experiencing both healthy and pathological aging. In addition, ERP modulations were reported in healthy elderly individuals after they received cognitive training, in line with neuroimaging studies showing reduced cortical activity in healthy elderly subjects after a WM training session (Brehmer et al., 2012; Heinzel et al., 2014).

Notably, to the best of our knowledge, no studies have examined resting-state EEG brain oscillation after WM training in healthy older adults. To fill this gap, we developed a 2 year research protocol to advance our understanding of the link between the cognitive and neural changes induced by WM training and, thus, on the mechanisms underlying WM cognitive training in older adults. Indeed, this latter topic is a critical aspect that is still missing from the aging literature.

The present study aimed to examine training-specific gains among healthy older adults immediately after WM training (short-term effect) and 6 months after it (long-term, follow-up, or maintenance effect). The well-validated WM training procedure in the study by Borella et al. (2010, 2017b) was conducted with a sample of healthy older adults. The specific training gains were assessed using a verbal WM criterion task closely similar to the one used in the training, which is the Categorization Working Memory Span task (CWMS; for the computerized version, see Spironelli et al., 2020). This WM task can also be used to assess intrusions errors, i.e., memory errors associated with the recall of non-target words, which represent a measure of inhibitory control failure (see Borella et al., 2007; Robert et al., 2009). Therefore, we also examined whether any transfer effect occurred for this measure.

Together with behavioral CWMS data, we analyzed a psychophysiological index of oscillatory responses, including the mean amplitude high-beta/alpha ratio, from 3 min resting-state EEG data (eyes open) collected before and after the WM training in a group of older adults, i.e., the training group (TG), and compared the data with those of a group engaged in alternative activities, i.e., the active control group (ACG), in the same period, who performed cognitive assessments. With respect to electrophysiological data, according to the study of Laufs et al. (2003), alpha activity is reduced attentively by external stimulus processing during resting state or when one is performing intentional mental operations with high cognitive load. In detail, decreased alpha power is associated with frontal and parietal activations of cortical structures involved in goal-directed cognition and behavior. In addition, the beta band characterizes the spontaneous cognitive activation during conscious rest. In the study by Oakes et al. (2004), the cerebral activity of participants at rest was measured simultaneously with PET-FDG and EEG. Their results revealed a significant correlation between the two techniques, showing that high-frequency EEG bands, i.e., high-beta and gamma waves, were positively correlated with higher regional glucose metabolism. On the contrary, the lower alpha band (8.5–10 Hz) revealed a clear negative correlation with brain metabolism, thus supporting the traditional interpretation of alpha rhythm as a physiological index inversely related to brain activation. The authors interpreted these results as direct (high-frequency EEG bands) or inverse (lower alpha EEG band) measures of neural activation in a resting-state condition (Oakes et al., 2004). For this reason, the high-beta/alpha ratio combines both the inhibitory component measured by alpha EEG and the activation component represented by the high beta into one measure, with the further advantage of statistically normalizing these measures across participants (the ratio between the two measures within-subjects). Concerning the localization of the expected changes induced by WM training, according to a large literature base involving healthy subjects (e.g., Emch et al., 2019), the frontal lobes play an important central role in WM, together with other secondary posterior regions. In addition, patients with lesions in their prefrontal cortex typically have impaired WM (e.g., Stuss et al., 1997; Jolly et al., 2020). Therefore, we expected that when using the described high-beta/alpha EEG ratio, prefrontal EEG sites would be the main neural hubs subjected to WM-induced plastic changes.

In line with previous studies (e.g., Borella et al., 2017b), we expected both short- and long-term specific training gains in the criterion task at the behavioral level, with TG (but not ACG) improving their WM performance. We also expected WM training to produce a transfer effect in the inhibitory control index, with TG, as compared with ACG, decreasing the intrusion errors they committed in the CWMS task. At the electrophysiological level, we expected no between-group differences in spontaneous EEG oscillatory activity before the WM training but significantly greater oscillatory responses in TG rather than ACG participants after the WM training, particularly in frontal brain sites. Indeed, according to the review of Constantinidis and Klingberg (2016), WM training increases prefrontal cortex activity. Whether WM training enhances the WM performance of older adults and, simultaneously, their resting-state neural oscillations, it is associated with the support of plasticity and the coordination of both information transfer within the brain and other important cognitive functions. Thus, we hypothesized that WM training actively shapes the power of spontaneous brain oscillations.

## Methods

### Participants

Healthy older adults were recruited by word of mouth and at social clubs for elderly people, according to the following inclusion criteria: (i) age between 64 and 75 years; (ii) Italian as their mother tongue; (iii) right-handed, as ascertained by the Edinburgh Handedness Inventory (Oldfield, 1971); (iv) a score of 27 or more in the Mini-Mental State Examination (MMSE; Folstein et al., 1975), which indicated a good cognitive functioning and no dementia or cognitive impairment; (v) good physical and mental health and normal or corrected-to-normal vision. Among the 24 volunteers for the study, all fulfilled the inclusion criteria.

Participants were randomized into two groups: the training group (TG), which attended the WM training, and the active control group (ACG). Six months after the training, all of the participants were called back for a follow-up session. All 24 older adults (see Table 1 for their descriptive statistics) elected to complete the whole neuropsychological and EEG assessment, including the pre-training, post-training, and follow-up sessions. None of the 24 participants dropped out during the whole study.

**Table 1:** Means (*M*) and standard deviations (± *SD*) of the demographic characteristics, cognitive functioning, and mood measures at pre-training for the training group vs. active control group (ACG) participants.

	**Training group** ***N*** **=** **12 (10 women)**		**Active control group** ***N*** **=** **12 (9 women)**		***t*(22) tests**
	***M***	***SD***	***M***	***SD***	
**Sociodemographic characteristics**					
*Age (years)*	68.83	±2.85	69.16	±3.18	−0.26
*Education (years)*	11.25	±2.14	11.00	±2.89	−0.24
*Handedness (Oldfield)*	99.33%	±2.31%	100.00%	±0.00%	1.00
**Cognitive functioning**					
*Mini-Mental State Examination*	27.75	±1.02	28.32	±1.15	−1.27
*Vocabulary*	53.92	±8.24	51.08	±11.35	0.70
*Ability to Solve Problems in Everyday Life*	11.95	±1.49	12.82	±1.13	−1.59
**Mood**					
*Geriatric Depression Scale*	1.75	±1.36	2.08	1.97	−0.48

All of the participants performed above the critical cut-off for their age and education in the Vocabulary test taken from the Wechsler Adult Intelligence Scale-Fourth Edition (WAIS-IV) (Wechsler, 2008; Italian norms by Orsini and Pezzuti, 2013) and in an objective performance-based measure of everyday functioning, specifically the Ability to Solve Problems in Everyday Life test (APE; Italian adaptation of the Everyday Problems Test; Borella et al., 2017a). Moreover, none of them reported signs of depression, as assessed with the Geriatric Depression Scale (GDS; Yesavage et al., 1982-1983).

The present study was approved by the Ethics Committee for Psychological Research—University of Padova (protocol number 3180). All of the participants gave written informed consent to participate in the study, which was performed in accordance with the ethical standards established in the Declaration of Helsinki (World Medical Association, 2013), and they were paid back for their travel costs to attend the experiment.

### Study Design

The study was conducted using a double-blind design. The assessment sessions were conducted by two experimenters, who knew that participants would have been involved in different activities and sessions, with only one group having attended WM cognitive training. They were unaware of the allocation of the participants into the training or control groups, and they did not attend any of the sessions. These two experimenters were previously trained on EEG collection and task administering. The training and alternative activity sessions for the TG and ACG, respectively, were run by a third experimenter, who previously was trained on managing the training protocol and the activities of the control group.

The participants were told about the aims of the study at the very end of the data collection (at follow-up). For ethical reasons, participants who were assigned to the control condition were offered to undergo the training program.

**Figure 1:**
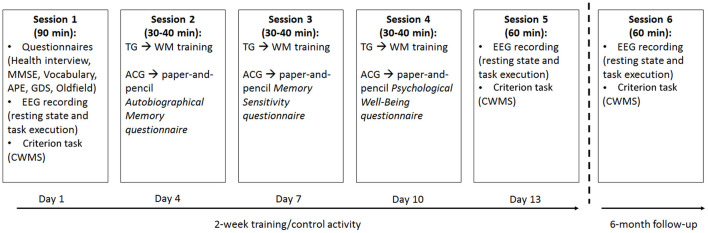
Schematic representation of the 2-week training/alternative activity sessions assigned to the training group (TG) and active control group (ACG) participants, as well as the 6-month follow-up. TG: Trained Group; ACG: Active Control Group; MMSE: Mini Mental State Examination; GDS: Geriatric Depression Scale; CWMS: Categorisation Working Memory Span Test; APE: Ability to Solve Problems in Everyday Life test.

All participants attended six individual laboratory sessions (Figure 1). The first and fifth were for the pre- and post-tests, and the sixth was the 6-month follow-up.

During the three assessment sessions, lasting about 90 min (pre-test) and 60 min (post-test and follow-up), respectively, the participants completed a battery of tasks: the CWMS -criterion- task (administered during the assessment sessions) was presented via computer to allow EEG collection. The Edinburgh Handedness Inventory, Health Interview, MMSE, Vocabulary, APE, and GDS were all paper-and-pencil tests administered only at pretesting (Figure 1).

During the other three sessions (2–4), lasting about 30–40 min each and completed within a 2 week time frame with a fixed 2 day break between each session, the TG has given the WM training, whereas the ACG was occupied in alternative activities (Figure 1). The duration of the training and alternative activities as well as the amount of interaction with the experimenter were much the same for the two groups.

In the practice phase, all of the participants were asked if they could see and hear the stimuli easily. They perceived the visual stimuli adequately. The volume of training stimuli was adjusted according to the preferences of each individual, and a sound amplifier for PCs was used. Therefore, after the practice phase, all of the participants were able to see and hear all of the stimuli administered in each task.

### Criterion Task (All Participants)

The computerized version of the CWMS (Spironelli et al., 2020), as in its original version (De Beni et al., 2008), consisted of 10 sets of word lists, which included 40 lists of five medium- to high-frequency words (divided into groups containing from two to six lists of words; two sets for each length). Among the total number of words in the task (200), 28% were animal words, and lists could contain zero, one, or two animal nouns, present in any position, including the last one. The participants were required to read each word appearing in the center of the computer screen and to press the spacebar whenever an animal noun appeared (processing phase). At the end of each set, when a triangle appeared in the center of the screen, the participants had to recall the last word on each list in serial order (maintenance phase), i.e., they needed to remember from two to six words, depending on the length of the set. Two practice trials containing two words to remember were given before the experimental task started.

The participants have visually presented the words on the computer screen as follows: black words were displayed in bold Courier New font (size: 24 points) in the center of the white screen for 2,000 ms, and the interval between each trial within the same set was 2,000 ms. The end of a list was signaled by a visual triangle (shown for 1,000 ms) presented together with a 1,000 Hz sound (presented for 200 ms). The experimenter wrote down recalled words on a dedicated form. To ensure that the participants were not trading off between processing the animal nouns and remembering the last words in the lists, 85% accuracy was required on the secondary task, i.e., pressing the spacebar whenever an animal noun appeared. All of the participants satisfied this criterion.

The total number of correctly recalled words was used as the measure of WM performance (maximum score = 20), which was considered the specific training gain. The words recalled incorrectly, i.e., recall of non-target words, were also computed and used as a measure of the inhibitory control of participants over no longer relevant information in WM, which was considered a transfer effect.

Two parallel versions of this task, wherein each one including five sets of word lists (one set for each length), were created and administered, one at pretest and the other at posttest, in a counterbalanced fashion across testing sessions. The pretest version was then presented at the 6-month follow-up.

### WM Training for the Training Group

The training task was presented individually in an auditory manner, adjusted to the hearing level of each participant to limit the influence of sensory variables on the outcomes. All of the verbal stimuli were presented by using a sound amplifier during the training sessions. The task consisted of a modified version of the CWMS task (see Borella et al., 2010), in which lists of audio-recorded words were presented and the participants were asked to recall target words and also to tap on the table with their hand when they heard the name of an animal.

The maintenance demand of the CWMS training task was manipulated by using an adaptive procedure in the first training session, i.e., the difficulty of the task increased based on whether a participant was successful at a given level, if not, the lowest level was presented. The demands of the task also varied and, depending on the session, could involve having to recall words proceeded by a beep (second training session) or an alternative recalling of the last or first word in each list (third training session). The processing demand (tapping on the table when an animal name was heard) was manipulated by varying the frequency of these animal words in the lists (second training session). This type of training procedure combined an adaptive procedure in the first training session with a standard one (from the easiest to the hardest trials) and was referred to as a hybrid procedure, which was considered to promote transfer effects (Borella et al., 2010).

### Activities for the Active Control Group

The participants in the ACG underwent the same number of individual sessions as the TG did, but they were asked to fill in the following paper-and-pencil questionnaires. Questionnaires included the Autobiographical Memory Questionnaire (De Beni et al., 2008), which entailed remembering common events related to their childhood, adulthood, and recent events and to rate their vividness; the Memory Sensitivity Questionnaire (De Beni et al., 2008), in which participants had to rate the frequency of behaviors dedicated to saving memories of life events; the Psychological Well-Being Questionnaire (De Beni et al., 2008), to rate the personal satisfaction of the participants with their life (past, present, and future), emotional competencies (ability to understand the emotions of their own and the others), and coping strategies regarding everyday problems.

### Data Recording and Analysis

The electrophysiological activity was recorded with 38 tin electrodes mounted on an elastic cap (Electro-Cap International Inc., Eaton, OH, USA) and positioned according to the International 10–20 system (Oostenveld and Praamstra, 2001). All of the cortical sites were referred to Cz during EEG acquisition and re-referenced off-line to the mean activity of the whole scalp by the average reference procedure. The data were stored using the NeuroScan software, version 4.1. The amplitude resolution was.1 μV, and the bandwidth ranged from DC to 100 Hz (6 dB per octave). The sampling rate was set at 500 Hz, and the impedance was kept below 5 KΩ (further details in Spironelli et al., 2020). After the data collection, the EEG signals were corrected for blinking and eye-movement artifacts, according to the eye movement modeling approach of Ille et al. (2002). All of the EEG data were divided into 2,048-ms time intervals. Indeed, given the constraint of the Brain Electrical Source Analysis (BESA) software to use 2^n^ samples, we needed to force the width of each interval to 1,024 samples, corresponding to a 2,048-ms interval. Each resting-state EEG recording, i.e., at pre-training, post-training, and follow-up at 3 min each, was divided into 2,048-ms time intervals. The continuous EEG data were transformed into the time-frequency domain using a fast Fourier transform (FFT), every task included 120 samples with.488 Hz FFT resolution. An artifact-rejection procedure was performed during each interval, with both amplitude and derivative thresholds (with respect to time) of 250 μV and 100 μV/ms, respectively. The remaining epochs were also inspected visually to remove any residual artifacts. On average, 89.93% of the epochs were accepted, equally distributed among sessions [pre-training: 88.75%, post-training: 90.94%, and follow-up 90.09%; *F*_(2, 44)_ = 0.55, *p* = 0.58, $\eta_{\text{p}}^{2}$ = 0.02]. After windowing each interval with a tapered cosine, the FFT was averaged across the epochs that were finally free of residual artifacts. In the following step, the EEG amplitude was normalized within each electrode as the contribution of each band to the whole 0.488–100 Hz spectral range and expressed as a percentage. Normalization allowed us to quantify the relative contribution of each EEG band with respect to total spectral power (% value) in the two main groups (TG vs. ACG) and to compare the same scalp locations in all samples.

For statistical purposes, we calculated a new physiological index as the mean amplitude of high-beta/alpha ratio^1^. We decided to consider the ratio between the two EEG bands, rather than the alpha or high-beta rhythms separately because the high-beta/alpha ratio combines both the inhibitory component and the activation component in one measure as measured by alpha EEG and as represented by the high-beta rhythm, respectively (Oakes et al., 2004). This had the further advantage of normalizing the measures statistically across participants, i.e., the ratio between the two measures by subject.

Electrodes were grouped into four clusters with two spatial factors, consisting of two levels each, namely anterior-posterior asymmetry and laterality. Therefore, each quadrant included the average amplitude of five electrodes, including anterior left (AL: F9-F7-FT7-F3-FC3), anterior right (AR: F10-F8-FT8-F4-FC4), posterior left (PL: CP3-P3-P7-TP7-O1), and posterior right (PR: CP4-P4-P8-TP8-O2).

For the demographic and cognitive tests, separate between-groups Student's *t-*tests were carried out on age, education, handedness, MMSE, Vocabulary WAIS-IV subscale, APE, GDS, CWMS accuracy performance, and CWMS intrusion errors at the pretest to control for any difference at the pretest stage. Gender distribution was analyzed using the non-parametric χ^2^ test.

Because of the small sample size, and as commonly done in cognitive training studies on aging (i.e., Borella et al., 2017a), Cohen's *d* values (1988), expressing the effect size of the comparisons, were computed within each group to better capture and assess the extent of the immediate (between the pre- and post-test sessions) and maintained (between the pre- and follow-up sessions) training gains. Values were corrected using the Hedges and Olkin (1985) correction factor to avoid the small sample bias. For the ANOVA results (see text footnote 2).

For the EEG data, we carried out an omnibus ANOVA including the between-subjects factor group (two levels, TG vs. ACG) and three within-subject factors, including session (three levels, pre-training vs. post-training vs. follow-up), region (two levels, anterior vs. posterior), and laterality (two levels, left vs. right hemisphere). Because the number of participants in each group was small (*n* = 12), we recognized that this analysis must be considered preliminary to ascertain whether the session factor showed a main effect or an interaction. Once this exploratory analysis revealed an effect of session, separate ANOVAs were carried out for each session, i.e., pre-training, post-training, and follow-up, on resting-state beta/alpha index that included the between-subjects factor group (two levels, TG vs. ACG) and two within-subject factors: region (two levels, anterior vs. posterior) and laterality (two levels, left vs. right hemisphere). Tukey's honestly significant difference (HSD) test was used to make *post hoc* comparisons (*p* < 0.05). In agreement with the behavioral data analysis, the Cohen's *d* (1988) effect sizes were also computed within each group.

In addition, Spearman's correlation analyses were carried out separately for TG and ACG participants, to ascertain whether post-training changes to the high-beta/alpha index at rest were significantly associated with better post-training performance on the CWMS task. Positive correlations marked those individuals with higher post-training scores on the CWMS task and higher post-training oscillatory responses at rest.

## Results

### Socio-Demographical and Behavioral Data

As can be seen in Table 1, the two groups did not differ significantly in age, gender distribution, education level, handedness, or the general cognitive functioning (MMSE, vocabulary, APE) and mood state measures.

As for the CWMS (see Table 2 for descriptive statistics), the groups did not differ at the pretest session in terms of either the CWMS score, representing the specific training gain, or intrusion errors, as an index of the efficiency of inhibitory control in the CWMS, representing a transfer effect [*t*_(22)_ = 0.38, *p* = 0.70 and *t*_(22)_ = 0.47, *p* = 0.68, respectively].

**Table 2:** Means (*M*) and standard deviations (±*SD*) of the criterion task (CWMS) and CWMS intrusion errors by group and assessment session.

	**Pre-training**				**Post-training**				**6-month follow-up**			
	**TG**		**ACG**		**TG**		**ACG**		**TG**		**ACG**	
***CWMS***	***M***	***SD***	***M***	***SD***	***M***	***SD***	***M***	***SD***	***M***	***SD***	***M***	***SD***
*Accuracy*	12.67	±2.96	12.08	±4.27	14.92	±2.39	13.17	±3.29	14.42	±2.53	13.00	±3.13
*Intrusion errors*	2.17	±1.19	1.83	±2.12	1.42	±0.90	1.50	±1.09	1.25	±1.05	2.08	±1.50

*CWMS, Categorization Working Memory Span test*.

Concerning the Cohen's *d* effect sizes, considering the specific training gain, i.e., the CWMS performance^2^, we found large to medium effect sizes immediately after the training and at the follow-up (0.80 and 0.61, respectively) for TG, whereas small effect sizes emerged for the ACG (0.27 and 0.24, respectively). Regarding the intrusion errors^2^, which account for transfer effects, the medium to nearly large effect sizes immediately after the training and at the follow-up (0.68 and 0.78, respectively) was for the TG, whereas small effect sizes emerged for the ACG (0.19 and 0.13, respectively).

### Electrophysiological Data

The preliminary omnibus ANOVA including all factors revealed a main effect of session [*F*_(2, 44)_ = 5.01, *p* < 0.05, $\eta_{\text{p}}^{2}$ = 0.19, Cohen's *d* = 0.95], and allowed us to carry out separate ANOVAs on resting-state high-beta/alpha index for each session, i.e., pre-training, post-training, and follow-up.

**Figure 2:**
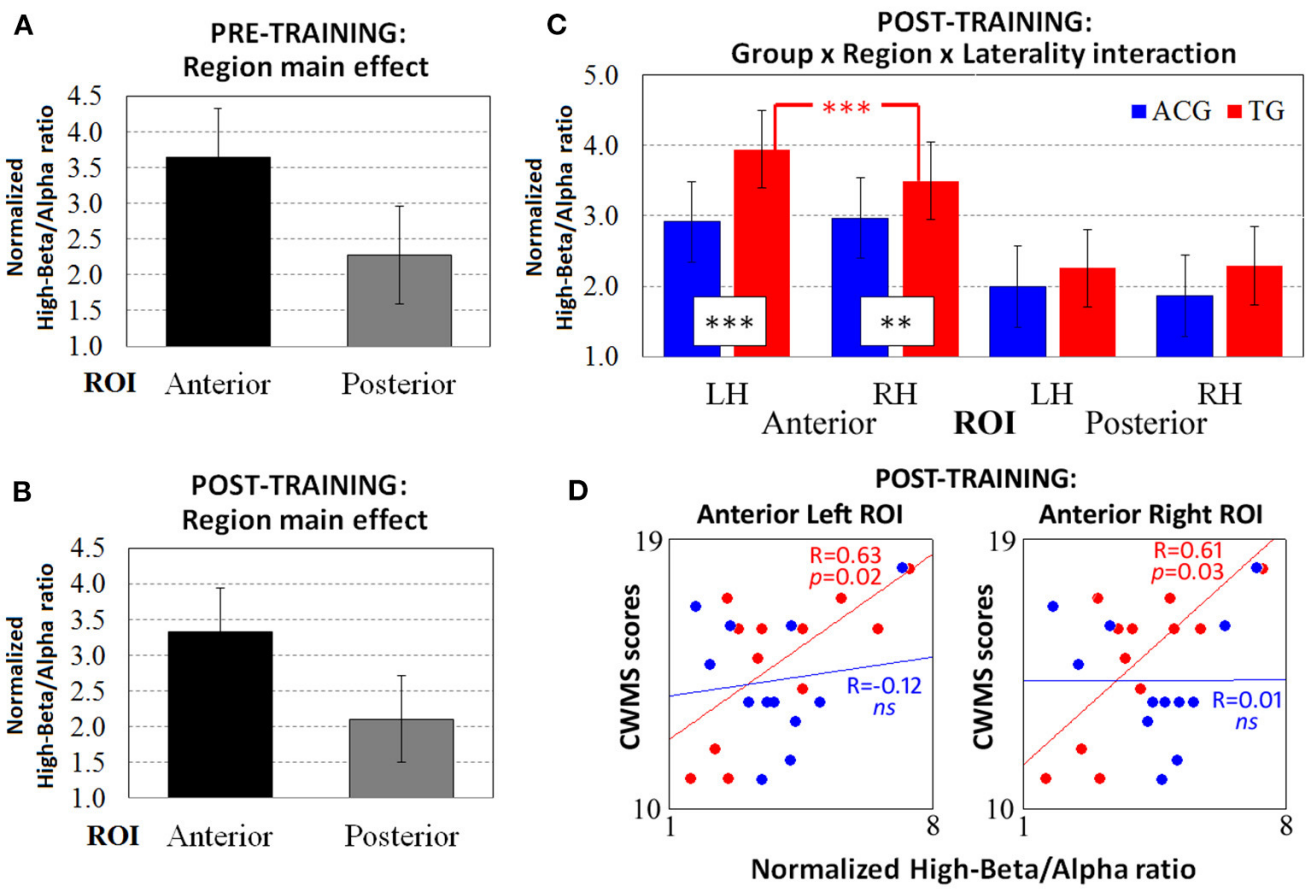
The resting-state normalized high-beta/alpha index from the pre-training EEG data revealed only **(A)** the main effect of the region. ANOVA on the post-training EEG data showed both **(B)** the main effect of region and **(C)** the significant three-way Group × Region × Laterality interaction. The Spearman correlations between high-beta/alpha indices on both anterior ROIs and CWMS post-training performance **(D)** were significant for TG participants (red dots and lines) but not for ACG participants (blue dots and lines). ***p < 0.001, **p < 0.01 Tukey HSD *post-hoc* comparisons. TG: Trained Group; ACG: Active Control Group; LH: Left Hemisphere; RH: Right Hemisphere; CWMS: Categorisation Working Memory Span Test.

The ANOVA carried out on the pre-training high-beta/alpha index revealed a significant main effect of region factor [*F*_(1, 22)_ = 33.53, *p* < 0.001, $\eta_{\text{p}}^{2}$ = 0.60, Cohen's *d* = 2.47], with greater anterior than posterior oscillatory activity (Figure 2A). No significant main effects or interactions were found with group factor.

The ANOVA carried out on the post-training high-beta/alpha index revealed a significant main effect of region factor [*F*_(1, 22)_ = 38.86, *p* < 0.001, $\eta_{\text{p}}^{2}$ = 0.63, Cohen's *d* = 2.66], same as for the pre-training resting-state session (Figure 2B). However, the three-way Group × Region × Laterality interaction [*F*_(1, 22)_ = 7.41, *p* = 0.01, $\eta_{\text{p}}^{2}$ = 0.25, Cohen's *d* = 1.16] showed different patterns of oscillatory activity in TG vs. ACG participants. Indeed, on both anterior ROIs (all *p*s < 0.01), the TG participants had a higher high-beta/alpha index than the ACG participants did (Figure 2C). In addition, the TG participants showed significantly greater left vs. right high-beta/alpha ratio in anterior ROIs (*p* < 0.001), with the amplitude of left clusters being significantly increased in left vs. right sites, whereas the ACG participants exhibited a bilateral pattern of high-beta/alpha oscillatory activity.

Spearman's correlations were computed between the post-training scores achieved in the CWMS task and the high-beta/alpha ratio indices obtained at left and right anterior ROIs (Figure 2D). The ACG showed no significant association between left and right physiological indices and CWMS post-training scores (all *p*s > 0.05), whereas the TG showed a significant positive correlation between the scores obtained for the CWMS task and both the anterior left [*R*_(10)_ = 63, *p* = 0.02] and right [*R*_(10)_ = 0.61, *p* = 0.03] high-beta/alpha indices. This indicated that the better the performance on the CWMS task after the training, the greater the oscillatory responses in the frontal sites at rest. Regarding the effect sizes of the correlation coefficients in the anterior left and right ROIs, Cohen's *d* was 0.86 and 0.7, respectively.

**Figure 3:**
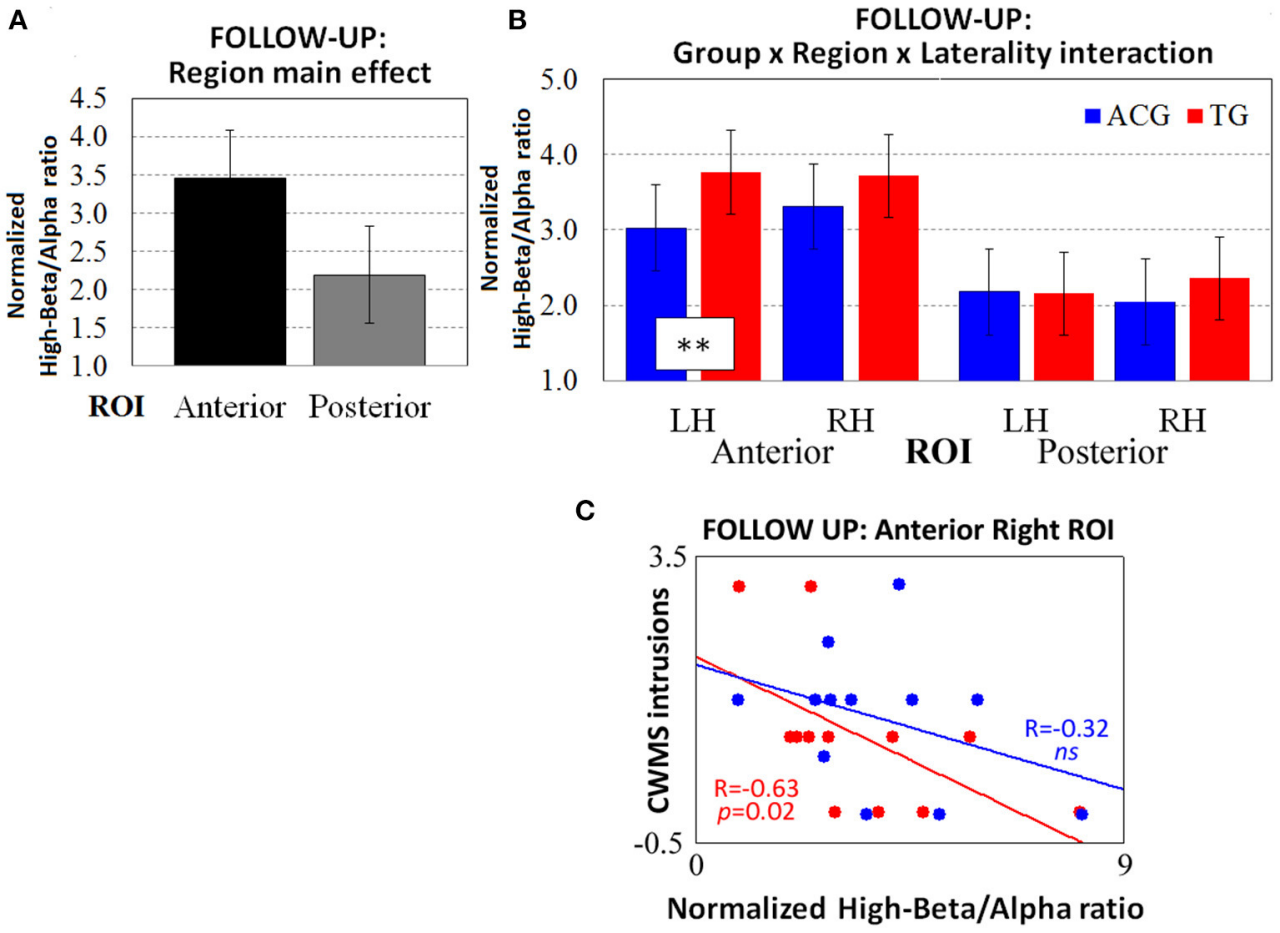
The resting-state normalized high-beta/alpha index from the follow-up EEG data revealed **(A)** the main effect of region and **(B)** the three-way Group × Region × Laterality interaction (*tendency*). The Spearman correlations between high-beta/alpha indices on anterior right ROIs and CWMS follow-up intrusions **(C)** were significant in TG participants (red dots and lines) but not in ACG participants (blue dots and lines). **p < 0.01 Tukey HSD *post-hoc* comparisons. TG: Trained Group; ACG: Active Control Group; LH: Left Hemisphere; RH: Right Hemisphere; CWMS: Categorisation Working Memory Span Test.

The ANOVA carried out on the follow-up high-beta/alpha index revealed a significant main effect of the region factor [*F*_(1, 22)_ = 39.21, *p* < 0.001, $\eta_{\text{p}}^{2}$ = 0.64, Cohen's *d* = 2.67], as it did in the previous session (Figure 3A). Furthermore, the three-way Group x Region x Laterality interaction was marginally significant [*F*_(1, 22)_ = 4.01, *p* = 0.05, $\eta_{\text{p}}^{2}$ = 0.25, Cohen's *d* = 0.85], revealing that, 6 months after the end of the WM training, the TG participants had higher oscillatory activity than the ACG participants on anterior left ROIs only (*p* < 0.01; Figure 3B).

The Spearman's correlations showed no significant association between left and right high-beta/alpha ratio indices and CWMS post-training scores in the ACG (all *p*s > 0.05), while showing a negative correlation between the follow-up intrusions of the TG in the CWMS task and the follow-up high-beta/alpha index on right anterior ROIs [*R*_(10)_ = −0.63, *p* = 0.02). This indicated that the lower the number of CWMS intrusions 6 months after the training, the greater the high-beta/alpha oscillatory activity in frontal right sites at rest (Figure 3C). Cohen's *d* was 0.41 in considering the effect size of the correlation coefficients (TG vs. ACG) on anterior right ROIs.

## Discussion

The present pilot study was aimed at investigating the effects of WM training on behavioral and spontaneous resting-state EEG changes in healthy older adults. In particular, we examined WM-specific training gains and a transfer effect, i.e., the ability to inhibit information in WM that is no longer relevant, at both the behavioral and brain levels. Past research has revealed the efficacy of the WM training procedure used here at the behavioral level (e.g., Borella et al., 2010, 2017b), but no studies have been carried out yet considering its effects (and even the effects of WM training more generally) on spontaneous cortical functioning. Most of the past research was aimed at studying the effects of training(s) on neural activity, as collected by EEG, while participants executed training(s) (e.g., Anguera et al., 2013). Such data show how various kinds of training affect brain oscillations but provide limited information on training effects/benefits beyond the training itself. In other words, generalization, or discussion of transfer effects, is not usually seen when EEG data refer to cognitive functioning directly associated with training. Although resting-state activity might be more difficult to interpret, it offers some important advantages when the experimental design is rigorous and different groups are compared. In further detail, resting-state activity measured before and after training may better reflect the plastic neural changes occurring after training and their persistent traces across time, which is a picture that can be masked during an active task. To the best of our knowledge, no studies have examined WM training changes in older adults by analyzing EEG oscillatory activity at rest. The present study was aimed at filling this gap, by analyzing a psychophysiological index of cortical activation, the mean amplitude high-beta/alpha ratio, in 3-min resting-state EEG data (eyes open) collected before and after WM training conducted with a group of older adults (TG) and comparing the data with an active control group engaged in cognitive assessments and alternative activities during the same period, rather than in the training, to ensure the best control sample for the TG. Finally, 6 months after the end of the WM training, all of the participants were called back for a follow-up assessment, which included electrophysiological resting-state recording. At least to our knowledge, no studies have examined maintenance effects regarding spontaneous oscillatory activity, and very few have analyzed such activity at the behavioral level.

Interestingly, in line with WM training studies in aging (Teixeira-Santos et al., 2019), the specific training gain was found only in the TG, as shown by the large effect size found for this group (as compared with the small effect size in the AG) in the criterion task, in the short term. In addition, a medium effect size was found for the specific training gains among the TG in the long term. This is in line with past studies using the same training regimen and, more in general, the very few WM training studies analyzing WM training-maintenance effects (i.e., Teixeira-Santos et al., 2019). As expected, these results confirmed the efficacy of WM training (and this specific procedure in particular) for improving the WM performance of elderly people, in both the short and long term. A transfer effect was also found regarding intrusion errors—as the index of the efficiency of inhibitory control—both at the posttest (medium effect size) and in the long-term (close to a large effect size). Such a transfer effect could be attributable to training activities that involve several processes, including the inhibition of no longer relevant information as well as attention shifting, to handle the different demands required by training tasks (see Borella et al., 2010, 2013). Notably, the larger effect sizes found in the long term, i.e., at the 6-month follow-up, as compared with the short-term ones, can indicate that more time is need for WM training to foster the ability to resist no longer relevant information. Indeed, a previous study using this same training procedure (Borella et al., 2017b) found a similar pattern of findings for intrusion errors, suggesting that certain abilities, like inhibitory abilities whose decline is particularly accentuated, thus not linear, with age take longer to benefit clearly from training activities. Notably, the ANOVA results did not show that the Group x Session interaction was significant for this measure. The divergent results between effect sizes and ANOVA may be due to the small sample size, which is one of the limits of the present study and causes the present study to be considered as a pilot one. However, the effect sizes indicating the presence of a transfer effect may subtend (a beginning of) changes that were more clearly found at the neural activity level but were still not explicit at the behavioral stage. Overall, these behavioral results both confirm that this WM training procedure fostered the specific gain and suggest the presence of a transfer effect on a mechanism (i.e., inhibition/attentional control) related to WM.

Regarding the electrophysiological data, preliminary analysis revealed the main effect of the session, allowing us to carry out a fine-grained analysis separately for the pre-training, post-training and, follow-up sessions. The analysis carried out on the normalized pre-training high-beta/alpha index revealed a main effect of region, suggesting that greater oscillatory responses appeared in frontal cortical sites, with no group differences. On the one hand, this analysis confirmed that the WM task used assesses WM and brain regions related to it (Constantinidis and Klingberg, 2016; Spironelli et al., 2020). On the other hand, it allows us to demonstrate that the training and the control group were similar, considering not only the inclusion criteria we set up a priori but also the baseline level of the cortical arousal of the participants in a resting-state condition. For this reason, post-training between-group effects reasonably could be associated with the assigned experimental condition, rather than with pre-existing differences. Greater oscillatory activity in the anterior cortical regions also was found in the post-training analysis. In addition, after the WM training, TG participants showed significantly greater oscillatory responses than ACG participants on both anterior ROIs. This result is in line with WM training studies showing that, after WM training, the regions involved in WM performance are the ones that changed (Constantinidis and Klingberg, 2016; Iordan et al., 2020). Interestingly, this increased cortical arousal was directly associated with better CWMS post-training performance: the higher the number of correct words recalled after the training, the greater the modulation of oscillatory responses in frontal sites at rest in only the TG participants. Thus, better management of WM task requests, probably due to the training activities, which led to improved WM performance, was related to greater frontal asymmetrical oscillatory activity in only the TG. This result supports the compensation hypothesis with aging (i.e., Cabeza et al., 2002), according to which aging brains recruit additional brain regions to “better” face task demands: the older adults in the TG who recruited more areas to compensate for losses had better WM performance than the ACG participants did. This pattern of findings also confirmed the role of the frontal region as an important locus for compensatory processes (Reuter-Lorenz and Park, 2014). Simultaneously, TG participants, but not ACG participants, showed higher oscillatory responses in left than in right anterior ROIs. This suggests that only the TG participants showed a shift from a more de-differentiated activity, i.e., the low specificity of processing, as shown during the pretest, to a more specific neural activity during the posttest. Therefore, the WM training could reduce the need to rely on compensatory neural mechanisms, thereby stimulating cortical efficiency. This pattern of findings is in line with other WM training studies involving older adults using fMRI (e.g., Buschkuehl et al., 2012). Indeed, laterality may serve as a marker of brain activity efficiency (Luo et al., 2016). A possible interpretation of our data could be that, because of the complexity of the WM task, a sort of over-activation compensated for age-related changes in neural efficiency during the pretest (i.e., Reuter-Lorenz and Cappell, 2008). The TG had more efficient behavioral and cortical functioning due to the training activities, as marked by greater and more specialized oscillatory activity.

Note that the leftward asymmetry found may also depend directly on the characteristics of the WM task used for training. The participants had to listen to a word list and then recall the last word of each string in serial order (maintenance phase) and tap on the table (or press the spacebar) whenever they recognized a word depicting an animal (processing phase), regardless of its position in the word list. Therefore, the requests of the CWMS test represent a challenging and complex condition, involving an in-depth analysis of words. Because language shows left hemisphere dominance for about 95% of right-handed individuals and 70–85% of left-handers (e.g., Knecht et al., 2000; Perlaki et al., 2013), the training itself probably stimulates the linguistic network, with the frontal operculum being important not only in articulation and phonological encoding (Paulesu et al., 1993; Indefrey and Levelt, 2004) but also in the hierarchical organization of high-level linguistic processes (e.g., Bookheimer, 2002; Hagoort, 2005). In addition, this area is sensitive to age changes when verbal material is used and appears to be critical for left hemisphere functioning because the frontal operculum is also involved in high-order cognitive functions that decline with aging, such as thinking, action planning, and goal-directed behaviors (Koechlin and Jubault, 2006). In any case, these results confirm that aging is characterized by a certain degree of plasticity in terms of neural reorganization, as elicited by the WM training activities, by increasing the range of neural activity of the WM circuitry (Iordan et al., 2020).

In line with this interpretation, as compared with the ACG participants, at the 6-month follow-up, the TG participants showed significantly greater oscillatory activity in frontal left ROIs only, but no within-group frontal asymmetry appeared. Therefore, the immediately increased cortical arousal at rest in frontal sites was still present 6 months after the training ended but only in the left anterior cluster of electrodes, confirming the increased oscillatory responses among the TG compared to the ACG. Thus, it seems that the pattern found at the posttest for the TG became clearer with time. Interestingly, considering the association with a behavioral performance at follow-up, no direct link with CWMS scores was found. However, a negative correlation with intrusion errors in the CWMS emerged. Again, the greater the cortical arousal in right frontal sites, the better the performance at follow-up, as revealed by the decrease in the number of intrusion errors at the CWMS test. This result regarding electrophysiological activity levels might indicate that more general processes could be at work in the long term, particularly lessened attentional control due to the more efficient ability to inhibit information that is no longer relevant from the WM (see Buschkuehl et al., 2012). It seems that in the long term, the WM task requires less attentional control and the training results when it comes to efficient processes, probably by making one's ability to suppress no longer relevant information more efficient, which is related to a clear shift from asymmetrical oscillatory activity to a “specific” one. This pattern of relationships (and changes at the cortical level) can also account for both the larger effect sizes and the asymmetrical EEG activity found, especially in the short term, for the TG. Thus, it was presumably no longer necessary in the long term to compensate at the cortical level to improve WM performance, but rather the spontaneous oscillatory activity could remain symmetrical because of changes in how one manages their attentional control resources, as suggested by the effect sizes found for intrusion errors. Although frontal regions had similar average levels of oscillatory responses as those in ACG participants did, the link with long-lasting gains was found for TG adults only.

Despite these interesting results, our study has some main limitations. The sample size was quite small, so this study must be considered as a pilot, and the use of a unique WM task to assess training gains could be another limitation. We did not use a hearing screening task, which could be recommended for auditory cognitive training. However, some precautions were adopted, such as the use of a sound amplifier, and the participants were asked if they could easily hear the training stimuli during the experimental sessions. Furthermore, the follow-up EEG data approached statistical significance, deserving careful interpretations, and generalizations. Future studies should confirm the present results and examine whether the same brain-activation patterns are found with other WM tasks and other cognitive tasks assessing transfer effects. Regarding the modulation in oscillatory activity found, including a group of young adults would have allowed us to better specify the nature of the present results with respect to their interpretation. It would also be of interest to examine training changes at the cortical level in older adults exhibiting cognitive impairment to better capture the value of this training procedure in counteracting aging changes and examine the degree of plasticity that this training can elicit. Nonetheless, the strengths of the study include its aim, the double-blind, repeated-measures experimental design, the presence of a 6-month follow-up session (rarely used in training studies, even those focusing on the behavioral data only), and the active control group. Furthermore, as stated at the beginning of the discussion, analyzing resting-state activity may offer some important advantages. In the present study, there were no between-group differences at baseline (during the pre-training condition), but we found significantly increased oscillatory activity after this WM training in frontal sites of only the TG participants. Considering that these two samples of older adults shared similar sociodemographic characteristics as well as comparable general cognitive functioning and mood symptoms, the results support the idea that the cortical-level changes of the TG could be reasonably attributed to their WM training activity.

In conclusion, the results of the present study provided further evidence that WM training is a promising procedure with which to sustain cognitive functioning in older adults, particularly by improving their WM performance, at least in the long term and in line with past studies carried out with the same training procedure (Borella et al., 2017b). In addition, resting-state EEG analysis showed, for the first time, that the WM training procedure used in this study increased frontal oscillatory activity at rest, revealing not only short-term but also long-term training effects on the cortical arousal of the TG participants. These results were closely associated with better WM performance and a decrease in intrusion errors over the long term. No such effects appeared in the ACG participants. Overall, these findings suggested that WM training represents a scaffold with which to counter the changes in older adults both in their cognitive and brain functioning. The results also indicated that WM training is a promising approach to foster the so-called functional cortical plasticity during aging, due to its ability to increase spontaneous oscillatory responses, particularly in frontal (left) brain regions.

## Data Availability Statement

The raw data supporting the conclusions of this article will be made available by the authors, without undue reservation.

## Ethics Statement

The studies involving human participants were reviewed and approved by the Ethics Committee for Psychological Research – University of Padova (Protocol number 3180). The patients/participants provided their written informed consent to participate in this study.

## Author Contributions

EB selected participants according to the inclusion criteria. CS acquired and analyzed the EEG data and drafted the figures. CS and EB drafted and revised the manuscript. Both authors contributed to the conception and design of the study, approved the final version of the work, and agreed to be accountable for all aspects of the work, to ensure that questions related to the accuracy or integrity of any part of the work are investigated and resolved appropriately.

## Funding

This work was funded by a grant from MIUR (Dipartimenti di Eccellenza DM 11/05/2017 no. 262) to the Department of General Psychology, University of Padova, and by the PRAT 2015 grant from the University of Padova, project no. CPDA152872 to CS.

## Conflict of Interest

The authors declare that the research was conducted in the absence of any commercial or financial relationships that could be construed as a potential conflict of interest.

## Publisher's Note

All claims expressed in this article are solely those of the authors and do not necessarily represent those of their affiliated organizations, or those of the publisher, the editors and the reviewers. Any product that may be evaluated in this article, or claim that may be made by its manufacturer, is not guaranteed or endorsed by the publisher.

## Footnotes

^1^We calculated the high-beta/alpha index as the ratio between the high-beta (20–35 Hz, effective β range 20.50–35.14 Hz) and alpha bands (8–2 Hz, effective α range 8.30–11.71 Hz).

^2^Separate ANOVAs were also carried out on CWMS performance - number of correctly recalled words- and intrusion errors. The ANOVA for the CWMS showed a main effect of the Session [*F*_(2, 44)_ = 6.78, *p* < 0.05, $\eta_{\text{p}}^{2}$ = 0.24] revealing a significant improvement from the pre- to the posttest (*Mdiff*. = 1.67, *p* = 0.01) and from the pretest to follow-up (*Mdiff*. = 1.33, *p* = 0.03). The main effect of the Group factor was not significant [*F*_(1, 22)_ = 1.15, *p* = 0.3], nor was the Group x Session interaction [*F*_(2, 44)_ < 1]. However, the planned comparisons based on our a priori hypotheses showed that only the TG showed a significant difference between the pretest and posttest (*Mdiff*. = −2.25, *p* = 0.02) and between the pretest and follow-up (*Mdiff*. = −1.75, *p* = 0.04). These findings revealed improvements in the CWMS score at both posttest and follow-up, which did not differ from each other. No significant differences were found in the ACG.Regarding the intrusion errors, the main effects of the Group [*F*_(1, 22)_ > 1] and Session factors [*F*_(2, 44)_ = 1.61, *p* = 0.21], as well as the Group x Session interaction [*F*_(2, 44)_ = 1.89, *p* = 0.16] were not significant. The planned comparison did not reveal any significant differences.
